# Optimal Sensor Placement in Buildings: Earthquake Excitation

**DOI:** 10.3390/s26082383

**Published:** 2026-04-13

**Authors:** Farid Ghahari, Daniel Swensen, Hamid Haddadi

**Affiliations:** 1 California Geological Survey, Sacramento, CA 95814, USA; 2 The B. John Garrick Institute for the Risk Sciences, University of California, Los Angeles, CA 90095, USA

**Keywords:** optimal sensor placement, buildings, beam model, Gaussian Process Regression, earthquake response

## Abstract

This study presents a methodology for determining the optimal placement of seismic sensors along the height of buildings to minimize the uncertainty in reconstructing structural responses at non-instrumented floors. Due to the extensive benefits of instrumentation—from model validation to damage detection and structural health monitoring—the number of instrumented structures is steadily increasing. However, to keep installation and maintenance costs within a reasonable range, structures are often instrumented sparsely. The response at non-instrumented locations is typically estimated using deterministic or probabilistic model-based, data-driven, or hybrid methods. Specifically, the authors recently proposed a method that combines a deterministic beam model with Gaussian Process Regression (GPR) to estimate responses at non-instrumented floors of an instrumented building. The present paper proposes a methodology to determine optimal sensor locations that minimize the uncertainty associated with this response estimation. This work is a sequel to a previous study that was limited to stationary excitation and extends the method to seismic excitations. The methodology is first verified through a numerical example and then applied to two real instrumented buildings. The results demonstrate that an average 40% reduction in uncertainty is achievable when sensors are positioned according to the proposed optimization approach, in comparison with a random distribution of sensors. Between the two real-life cases studied in this paper, the level of reduction in the response uncertainty is around 10% for the 52-story building because the existing sensors are almost uniformly distributed, while it is around 80% for the 73-story building because the existing sensors are distributed to measure the localized behavior of the building.

## 1. Introduction

With more than 1390 active stations (including ground-response stations, instrumented buildings, bridges, and other facilities), the California Strong Motion Instrumentation Program (CSMIP) is one of the most comprehensive seismic instrumentation programs in the world. Since its inception in 1972, the program has collected an extensive archive of ground and structural response records using more than 10,000 sensors. These data have been widely used by researchers and engineers to advance the understanding of structural seismic behavior and to improve seismic design guidelines [1,2] as well as post-earthquake structural health assessment [3].

Seismic design codes, when applicable, require installing a limited number of sensors at structures and not on all floors of the buildings. Also, due to the cost of instrumentation and maintenance, most of the CSMIP structures have not been densely instrumented. As a result, building responses are measured at only a limited number of floors. This sparsity can restrict the direct use of recorded seismic data for applications like post-earthquake damage assessment based on engineering demand parameters, such as interstory drift ratios. Although sophisticated finite element model-updating techniques (e.g., [4,5,6,7]) can predict responses at non-instrumented floors, these approaches are time- and labor-intensive and are not practical for large-scale regional programs like CSMIP. Interpolation methods, such as cubic splines, may appear to be a viable solution [8,9,10]; however, because these methods are deterministic, the associated prediction uncertainties remain unknown.

To develop a fast yet reliable method for estimating responses at non-instrumented floors, while also systematically quantifying uncertainty, we recently introduced a hybrid deterministic–probabilistic framework [11]. This method combines a coupled flexural–shear beam model [12]—calibrated by adjusting only a few parameters—with a Gaussian Process Regression (GPR) model [13] to predict structural responses at non-instrumented locations. In this approach, the beam model is first calibrated using data from instrumented floors, and the resulting residual errors are used to train the GPR model. This method is applicable to a broad inventory of buildings—not limited to beam-type structures—under various excitation levels, encompassing both linear and nonlinear behavior [11]. The mean response and its associated uncertainty at non-instrumented floors are subsequently obtained by integrating the deterministic prediction from the beam model with the probabilistic correction provided by the GPR model.

The level of estimation uncertainty depends on both the number and the distribution of sensors along the height of the building [14,15]. Following our previous work [11], a new project was initiated within CSMIP to determine the optimal sensor configuration that minimizes this overall uncertainty. Optimal Sensor Placement (OSP) has been an active research topic across various engineering fields [16,17,18], and its application in earthquake engineering was revitalized around 2000 [19,20,21,22,23,24]. While the present study builds on prior works that use information theory (e.g., [25,26,27]), the proposed OSP formulation differs in three key aspects:

- It is fully consistent with the hybrid response–reconstruction framework, providing a fast and practical end-to-end solution.
- The objective function explicitly targets minimizing response-estimation uncertainty, the primary goal for CSMIP and other strong motion instrumentation programs. This objective function has not received much attention in the literature, while it is crucial for programs like CSMIP.
- The method does not require detailed structural information or a numerical model of the building. With limited resources and many instrumented structures, it is crucial to have a method that works for a large inventory of buildings without needing detailed information and complicated engineering modeling and calculations.

This study extends our recent work [28], which addressed the OSP problem under the assumption of stationary (ambient) excitation. While the objective function remains similar, in the previous study, the GPR model was analytically derived using random vibration theory, based on the assumption of a beam-like structural behavior and stationary excitation. This study addresses these limitations—specifically modeling error, linearity, and stationarity—by employing a hybrid model-data approach. The beam model captures the global response, while the residual response is captured by a GPR model trained on recorded measurements.

In the next section, the two-step calibration procedure for the coupled beam–GPR model, which was originally developed in [11], is first reviewed, followed by a detailed presentation of the optimal sensor placement methodology. The performance of the proposed approach is then evaluated using a series of synthetic yet realistic examples for which the true responses at all floors are available. Finally, the method’s application to real buildings is demonstrated. The main findings of this study are summarized in Section 6.

## 2. The Model Calibration

**Figure 1:**
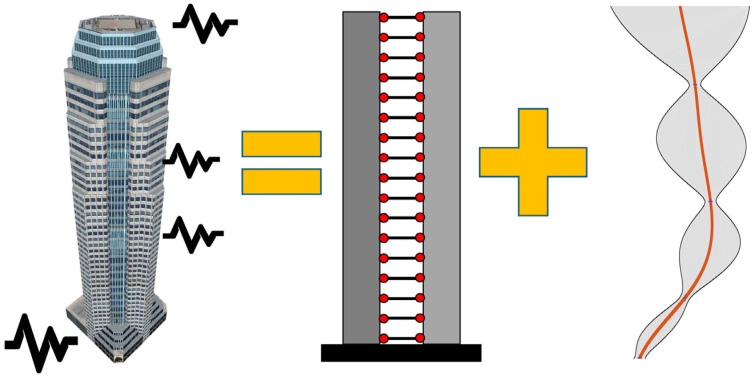
The modeling strategy used in this study. The structural response at any level is assumed to be a combination of a deterministic shear–flexural beam model and a probabilistic GPR model.

The model calibration approach proposed in [11] is schematically presented in Figure 1. It is assumed that the building response under earthquake excitation is available at $n_{d}$ locations $x_{i}$ along the height of the building. These time history responses, e.g., absolute accelerations, are denoted as $u_{m}\left[x_{i},n\right]$, where $i=1,\ldots ,n_{d}$ and n=1,…,N, with N being the number of time samples. It is assumed that the building can be represented as a combination of a deterministic shear-flexural beam model [12] and a probabilistic model represented by a GPR model. Therefore,

(1) $$u_{m}\left[x_{i},n\right]=u_{B}\left[x_{i},n;\theta \right]+f_{GPR}\left[x_{i},n;\vartheta \right]+\epsilon$$

where $u_{B}$ is the deterministic response predicted by the beam model and $f_{GPR}$ is the probabilistic correction predicted by the GPR model. ϵ is the measurement noise, which is assumed to be a zero-mean stationary white Gaussian random process with constant variance $\sigma_{0}^{2}$ at all channels.

The beam model serves as a backbone to ensure adherence to physics, preserving temporal correlations throughout the entire time-history. It is straightforward to calibrate, requiring only a few parameters (enclosed in θ), as briefly described in the next section, and is computationally efficient compared to detailed Finite Element models. This calibration is carried out first, and its results are not sensitive to sensor locations because the beam response at every location is sufficiently sensitive to all calibration parameters. Therefore, it does not directly influence the optimal sensor placement framework which is described later.

After completing the beam model calibration, the residual responses denoted as $y\left[x_{i},n\right]=u_{m}\left[x_{i},n\right]-u_{B}\left[x_{i},n;\theta \right]$ can be expressed as follows:

(2) $$y[x_{i},n]=f_{GPR}\left[x_{i},n;\vartheta \right]+\epsilon .$$

These residual responses are then used to find hyperparameters of the GPR model, ϑ. A summary of this process is provided later. Following the two-step model-data calibration process described above, the mean response at non-instrumented floors can be predicted using Equation (1) by combining the deterministic prediction provided by the beam model and the mean prediction provided by the GPR model. While the prediction uncertainty at instrumented floors is limited to the measurement noise, it can be large at non-instrumented floors and can be quantitively assessed using the GPR model variance together with the measurement noise variance.

### 2.1. Beam Model

The absolute seismic response of a shear-flexural beam model at a normalized height $x_{i}=h_{i}/H$ can be approximately calculated through the superposition of the first $n_{m}$ dominant modes as follows [29]:

(3) $$u_{B}\left[x_{i},n;\theta \right]≅u_{g}\left[n\right]+\sum_{k=1}^{n_{m}}\Gamma_{k}\varphi_{k}\left(x_{i}\right)q_{k}[n],$$

where $q_{k}\left[n\right]$ is the relative response of a Single-Degree-Of-Freedom (SDOF) system with undamped natural frequency $\omega_{k}$ and damping ratio $\xi_{k}$ to ground motion excitation $u_{g}\left[n\right]$. $\Gamma_{k}$ and $\varphi_{k}\left(x_{i}\right)$ are the k-th modal contribution factor and mode shape, respectively. $q_{k}\left[n\right]$ is calculated as the discrete convolution $q_{k}\left[n\right]=g_{k}^{d,a}[n]*a_{g}\left[n\right]$, where $a_{g}\left[n\right]$ is the discrete-time ground acceleration and $g_{k}^{d,a}[n]$ is the discrete-time Impulse Response Function (IRF) corresponding to relative displacement or acceleration, defined respectively as follows:

(4) $$g_{k}^{d}\left[n\right]=\frac{-∆t}{\omega_{d,k}}e^{-\xi_{k}\omega_{k}n∆t}\sin\left(\omega_{d,k}n∆t\right),$$

(5) $$g_{k}^{a}\left[n\right]=\frac{-∆t\omega_{k}}{\sqrt{1-\xi_{k}^{2}}}e^{-\xi_{k}\omega_{k}n∆t}\left[(2\xi_{k}^{2}-1)\sin\left(\omega_{d,k}n∆t\right)-2\xi_{k}\sqrt{1-\xi_{k}^{2}}\cos\left(\omega_{d,k}n∆t\right)\right],$$

with $\omega_{d,k}=\omega_{k}\sqrt{1-\xi_{k}^{2}}$ and ∆t being the sampling time. Note that Equation (3) can be used for any type of response (acceleration, velocity, and displacement) as long as the corresponding $u_{g}[n]$ and $q_{k}\left[n\right]$ are used. The analytical mode shape can be calculated as follows [12]:

(6) $$\varphi_{k}\left(x_{i}\right)=\sin\left(\gamma_{k}x_{i}\right)-\frac{\gamma_{k}}{\beta_{k}}\sinh\left(\beta_{k}x_{i}\right)+\eta_{k}\left[\cosh\left(\beta_{k}x_{i}\right)-\cos\left(\gamma_{k}x_{i}\right)\right]$$

with

(7) $$\eta_{k}=\frac{\gamma_{k}^{2}\sin\gamma_{k}+\gamma_{k}\beta_{k}\sinh\beta_{k}}{\gamma_{k}^{2}\cos\gamma_{k}+\beta_{k}^{2}\cosh\beta_{k}},$$

where $\beta_{k}^{2}=\alpha^{2}+\gamma_{k}^{2}$, α is a dimensionless parameter representing the shear to flexural contribution [12], and $\gamma_{k}$ is the k-th root of the following characteristic equation

(8) $$r\left(\alpha ,\gamma_{k}\right)=2+\left(2+\frac{\alpha^{4}}{\gamma_{k}^{2}{\beta_{k}}^{2}}\right)\cos\gamma_{k}\cosh\beta_{k}+\frac{\alpha^{2}}{\gamma_{k}\beta_{k}}\sin\gamma_{k}\sinh\beta_{k}=0.$$

Miranda and Akkar [30] recommend using the alternative forms of Equations (6)–(8), presented below, to prevent numerical issues for large values of α:

(9) $$\varphi_{k}\left(x_{i}\right)=\sin\left(\gamma_{k}x_{i}\right)-\eta_{k}\cos\left(\gamma_{k}x_{i}\right)+\frac{1}{2}\left(\eta_{k}-\frac{\gamma_{k}}{\beta_{k}}\right)e^{\beta_{k}x_{i}}+\frac{1}{2}\left(\eta_{k}+\frac{\gamma_{k}}{\beta_{k}}\right)e^{-\beta_{k}x_{i}}$$

(10) $$\eta_{k}=\frac{\gamma_{k}}{\beta_{k}}+\frac{P_{k}}{Q_{k}}=\frac{\beta_{k}\gamma_{k}^{2}\sin\gamma_{k}-\gamma_{k}^{3}\cos\gamma_{k}-\gamma_{k}\beta_{k}^{2}e^{-\beta_{k}}}{\beta_{k}\gamma_{k}^{2}\cos\gamma_{k}+\beta_{k}^{3}\cosh\beta_{k}},$$

(11) $$r\left(\alpha ,\gamma_{k},\beta_{k}\right)=4\gamma_{k}^{2}{\beta_{k}}^{2}e^{-\beta_{k}}+\left(\alpha^{4}+2\gamma_{k}^{2}{\beta_{k}}^{2}\right)\cos\gamma_{k}+\alpha^{2}\gamma_{k}\beta_{k}\sin\gamma_{k}+\left[\left(\alpha^{4}+2\gamma_{k}^{2}{\beta_{k}}^{2}\right)-\alpha^{2}\gamma_{k}\beta_{k}\sin\gamma_{k}\right]e^{-2\beta_{k}}=0.$$

The only parameter required to calculate mode shape is α which can vary from 0 (pure flexural behavior) to infinity (pure shear behavior) [31,32]. Once the mode shapes are obtained, the modal contribution factor is calculated as follows:

(12) $$\Gamma_{k}=\frac{I_{1}}{I_{2}}=\frac{\int_{0}^{1}\varphi_{k}\left(x\right)dx}{\int_{0}^{1}{\varphi_{k}\left(x\right)}^{2}dx}$$

where x, the normalized height parameter, continuously varies from 0 to 1. To use Equation (3), natural frequencies and modal damping ratios are needed. While modal damping ratios must be specified independently, it can be shown that the natural frequencies of higher modes can be computed from the fundamental natural frequency $\omega_{1}$ as follows [12]:

(13) $$\omega_{k}^{2}=\frac{\gamma_{k}^{2}{\beta_{k}}^{2}}{\gamma_{1}^{2}{\beta_{1}}^{2}}\omega_{1}^{2}.$$

To estimate these controlling parameters of the beam model, i.e., $\theta ={\left[\omega_{1},\alpha ,\xi_{1},\ldots ,\xi_{n_{m}}\right]}^{T}$, an input-output Bayesian estimation method [33] briefly reviewed below, is employed.

Assuming that the simulation error between measured and predicted responses follows a stationary zero-mean white Gaussian random process with diagonal covariance matrix $R=\sigma_{e}^{2}I_{n_{d}\times n_{d}}$, the likelihood of observing the data can be expressed as follows:

(14) $$p\left(y_{m}|\theta \right)=\prod_{n=1}^{N}\frac{1}{{\left(2\pi \right)}^{n_{d}/2}{\left|R\right|}^{1/2}}e^{-\frac{1}{2}{\left(y_{m}\left[n\right]-y_{B}\left[n\right]\right)}^{T}R^{-1}(y_{m}\left[n\right]-y_{B}\left[n\right])},$$

where $y_{m}\left[n\right]={\left[u_{m}\left[x_{1},n\right],\ldots ,u_{m}\left[x_{n_{d}},n\right]\right]}^{T}$ and $y_{B}\left[n\right]={\left[u_{B}\left[x_{1},n\right],\ldots ,u_{B}\left[x_{n_{d}},n\right]\right]}^{T}$ are the vectors of measured and beam-predicted responses, respectively. Assuming a Gaussian prior Probability Density Function (PDF) p(θ), the posterior PDF of the parameter vector can be expressed as follows:

(15) $$p\left(\theta |y_{m}\right)\propto p\left(y_{m}|\theta \right)p\left(\theta \right).$$

The Maximum A Posterior (MAP) estimate of θ is obtained by maximizing the exponent of the posterior PDF, that is,

(16) $$\hat{\theta }=\underset{\theta }{\max}\left\{-\frac{1}{2}\sigma_{e}^{-2}{\left(Y_{m}-Y_{B}\right)}^{T}(Y_{m}-Y_{B})-\frac{1}{2}{\left(\theta -{\hat{\theta }}^{-}\right)}^{T}{\left({\hat{P}}_{\theta }^{-}\right)}^{-1}\left(\theta -{\hat{\theta }}^{-}\right)\right\},$$

where $Y_{m}={\left[{y_{m}\left[1\right]}^{T},\ldots ,{y_{m}\left[N\right]}^{T}\right]}^{T}$ and $Y_{B}={\left[{y_{B}\left[1\right]}^{T},\ldots ,{y_{B}\left[N\right]}^{T}\right]}^{T}$, and ${\hat{\theta }}^{-}$ and ${\hat{P}}_{\theta }^{-}$ are the prior mean vector and covariance matrix of the parameter vector θ, respectively. The predicted response $Y_{B}$ is computed by running the beam model, which is a nonlinear function of the parameter vector, i.e., $Y_{B}=h(\theta )$. Using a first-order Taylor approximation around the prior mean, i.e., $Y_{B}\approx h\left({\hat{\theta }}^{-}\right)+H\left(\theta -{\hat{\theta }}^{-}\right)$, the solution to Equation (16) can be expressed as follows:

(17) $$\hat{\theta }={\hat{\theta }}^{-}+K\left[Y_{m}-h\left({\hat{\theta }}^{-}\right)\right],$$

where $H_{Nn_{d}\times n_{\theta }}={\left.\frac{\partial h(\theta )}{\partial \theta }\right|}_{\hat{\theta }={\hat{\theta }}^{-}}$ is the Jacobian matrix (see Appendix A) and K is the Kalman Gain matrix calculated as follows:

(18) $$K={\hat{P}}_{\theta }^{-}H^{T}{\left(H{\hat{P}}_{\theta }^{-}H^{T}+\sigma_{e}^{2}I_{Nn_{d}\times Nn_{d}}\right)}^{-1}.$$

The posterior covariance matrix is calculated as follows:

(19) $${\hat{P}}_{\theta }=\left(I_{n_{\theta }\times n_{\theta }}-KH\right){\hat{P}}_{\theta }^{-}.$$

This process can be repeated by replacing ${\hat{\theta }}^{-}=\hat{\theta }$ and ${\hat{P}}_{\theta }^{-}={\hat{P}}_{\theta }+Q$ where Q $\in R^{n_{\theta }\times n_{\theta }}$ is a diagonal matrix with small diagonal elements to accelerate convergence. Additionally, to avoid numerical difficulties when working with a large amount of data, the data can be divided into overlapping windows and the above formulation applied sequentially (see, e.g., [34]).

### 2.2. Gaussian Process Regression

In the second step, Gaussian Process Regression (GPR) [13], a Bayesian approach for function approximation, is used to estimate the posterior probability distribution of the residual responses at non-instrumented levels.

Let $\bar{x}={\left[\begin{array}{cccc} {\bar{x}}_{1} & {\bar{x}}_{2} & \cdots & {\bar{x}}_{n_{d}} \end{array}\right]}^{T}$ denote the normalized heights of the instrumented floors (an overbar is used to distinguish instrumented from non-instrumented levels), and let $\bar{y}[n]={\left[\begin{array}{cccc} {\bar{y}}_{1}[n] & {\bar{y}}_{2}[n] & \cdots & {\bar{y}}_{n_{d}} \end{array}[n]\right]}^{T}$ be the vector of noisy residuals at these locations. It is assumed that the residual response along the height of the building follows a GPR model $f\left(x\right)\sim GP\left(m_{0}\left(x\right),k\left(x,x^{\prime };\vartheta \right)\right)$, with prior mean $m_{0}\left(x\right)=E\left[f\left(x\right)\right]$ and prior covariance kernel $k\left(x,x^{\prime };\vartheta \right)=E\left[\left(f\left(x\right)-m_{0}\left(x\right)\right)\left(f\left(x^{\prime }\right)-m_{0}\left(x^{\prime }\right)\right)\right]$ where $E\left[.\right]$ denotes the expected value and ϑ is the vector of hyperparameters defining the covariance kernel. Accordingly, the collection of measured residuals and the predicted residual at a non-instrumented height $\hat{x}$, $f\left(\hat{x}\right)$, follows a joint Gaussian distribution given by (The time index n is dropped for simplicity).

(20) $$\left[\begin{array}{c} \bar{y}\\ f\left(\hat{x}\right) \end{array}\right]\sim N\left(\left[\begin{array}{c} m_{0}\left(\bar{x}\right)\\ m_{0}\left(\hat{x}\right) \end{array}\right],\begin{array}{cc} C\left(\bar{x},{\bar{x}}^{\prime };\vartheta \right)+\sigma_{0}^{2}I_{n_{d}\times n_{d}} & k\left(\bar{x},\hat{x};\vartheta \right)\\ {k\left(\bar{x},\hat{x};\vartheta \right)}^{T} & k\left(\hat{x},\hat{x};\vartheta \right) \end{array}\right),$$

where the covariance matrix $C\left(\bar{x},{\bar{x}}^{\prime };\vartheta \right)$ is calculated using the kernel function as follows:

(21) $$C\left(\bar{x},{\bar{x}}^{\prime };\vartheta \right)=\left[\begin{array}{cccc} k\left({\bar{x}}_{1},{\bar{x}}_{1};\vartheta \right) & k\left({\bar{x}}_{1},{\bar{x}}_{2};\vartheta \right) & \cdots & k\left({\bar{x}}_{1},{\bar{x}}_{n_{d}};\vartheta \right)\\ k\left({\bar{x}}_{2},{\bar{x}}_{1};\vartheta \right) & k\left({\bar{x}}_{2},{\bar{x}}_{2};\vartheta \right) & \vdots & k\left({\bar{x}}_{2},{\bar{x}}_{n_{d}};\vartheta \right)\\ \vdots & \cdots & \ddots & \vdots \\ k\left({\bar{x}}_{n_{d}},{\bar{x}}_{1};\vartheta \right) & k\left({\bar{x}}_{n_{d}},{\bar{x}}_{2};\vartheta \right) & \cdots & k\left({\bar{x}}_{n_{d}},{\bar{x}}_{n_{d}};\vartheta \right) \end{array}\right],$$

And the vector $k\left(\bar{x},\hat{x};\vartheta \right)$ represents the correlation between the measured residuals and the predicted residuals, i.e.,

(22) $$k\left(\bar{x},\hat{x};\vartheta \right)=\left[\begin{array}{c} k\left({\bar{x}}_{1},\hat{x};\vartheta \right)\\ k\left({\bar{x}}_{2},\hat{x};\vartheta \right)\\ \vdots \\ k\left({\bar{x}}_{n_{d}},\hat{x};\vartheta \right) \end{array}\right].$$

Therefore, the conditional distribution of the residual at the non-instrumented floor $\hat{x}$ is Gaussian with

(23) $$\left.f\left(\hat{x}\right)\right|\bar{x},\bar{y}\sim N\left(m_{f\left(\hat{x}\right)},\sigma_{f\left(\hat{x}\right)}^{2}\right),$$

where

(24) $$m_{f\left(\hat{x}\right)}={k\left(\bar{x},\hat{x};\vartheta \right)}^{T}{\left[C\left(\bar{x},{\bar{x}}^{\prime };\vartheta \right)+\sigma_{0}^{2}I\right]}^{-1}\bar{y},$$

(25) $$\sigma_{f\left(\hat{x}\right)}^{2}=k\left(\hat{x},\hat{x};\vartheta \right)-{k\left(\bar{x},\hat{x};\vartheta \right)}^{T}{\left[C\left(\bar{x},{\bar{x}}^{\prime };\vartheta \right)+\sigma_{0}^{2}I\right]}^{-1}k\left(\bar{x},\hat{x};\vartheta \right),$$

are the posterior mean and variance of the predicted residual at $\hat{x}$.

In the formula above, the prior mean is assumed to be zero. This is a common assumption in GPR applications and is consistent with the problem considered here, since the deterministic beam model captures the main trend of the response and the residual represents only the discrepancy. This stochastic correction, when added to the deterministic prediction obtained from the calibrated beam model, provides the total response at any non-instrumented floor at any time instant. In other words, the total response at a non-instrumented floor with normalized height $\hat{x}$ follows a Gaussian distribution with mean and variance given by the sum of the deterministic prediction and the GPR posterior statistics.

As described in detail in [11], the hyperparameters of the GPR kernel function, ϑ, can be obtained by maximizing the likelihood (or equivalently minimizing the negative log-likelihood) of the measured residuals shown in Equation (26), assuming a parametric representation of the kernel function:

(26) $$NLL\left(\bar{y}\right)=\frac{1}{2}{\bar{y}}^{T}{\left[C\left(\bar{x},{\bar{x}}^{\prime };\vartheta \right)+\sigma_{0}^{2}I\right]}^{-1}\bar{y}+\frac{1}{2}\log\left|\left(C\left(\bar{x},{\bar{x}}^{\prime };\vartheta \right)+\sigma_{0}^{2}I\right)\right|+\frac{n_{d}}{2}\log2\pi ,$$

In this study, a Squared Exponential (SE) kernel is used, defined as follows:

(27) $$k_{SE}\left(x,x^{\prime };\vartheta \right)=\sigma_{f}^{2}e^{-\frac{{\left|x-x^{\prime }\right|}^{2}}{2\sigma_{l}^{2}}},$$

where $\sigma_{f}^{2}$ and $\sigma_{l}$ are elements of the hyperparameter vector ϑ, representing the signal variance and the correlation length, respectively. These hyperparameters can, in principle, be adjusted independently at each time instant, as performed in [11]. However, since the purpose of this study is optimal sensor placement rather than response reconstruction, a fixed set of hyperparameters is used for the entire time history, as described later. It is noteworthy to mention that the SE kernel is selected in this study because it is the most widely adopted covariance function in the literature for modeling smooth physical processes, including seismic structural responses. Its ability to represent the underlying physics with minimal hyperparameters makes it a robust choice for this study. Furthermore, as this kernel was successfully employed in our previous work on response reconstruction [11], it was utilized here to maintain methodological consistency with those results.

## 3. Optimal Sensor Placement Strategy

Having established the method to predict the response of the building at non-instrumented floors and to quantify the associated uncertainty, the optimal sensor locations are determined such that the total prediction uncertainty is minimized, following the approach used by other researchers (e.g., [27]).

Therefore, the optimal normalized heights of $n_{d}$ sensors, denoted by the vector $x^{*}={\left[\begin{array}{cccc} {{\bar{x}}^{*}}_{1} & {{\bar{x}}^{*}}_{2} & \cdots & {{\bar{x}}^{*}}_{n_{d}} \end{array}\right]}^{T}$, are obtained by solving the following minimization problem:

(28) $$x^{*}=\underset{\bar{x}\in S}{\min}\int \left(\left.\sigma_{f\left(\hat{x}\right)}^{2}\right|\bar{x}\right)d\hat{x},$$

where $\bar{x}$ is the vector of instrumented heights, S is the collection of all feasible heights for instrumentation, and $\sigma_{f\left(\hat{x}\right)}^{2}$ is the posterior variance of the predicted response at non-instrumented levels, calculated using Equation (25). This objective function guarantees that if sensors are placed at the optimal locations and the hybrid model-data model is used for response prediction at non-instrumented floors, the level of total uncertainty is minimized.

For practical implementation, the objective function in Equation (28) can be expressed as a discrete summation:

(29) $$OF=\frac{1}{n_{0}}\sum_{i=1}^{n_{0}}\left.\sigma_{f\left({\hat{x}}_{i}\right)}^{2}\right|\bar{x},$$

where ${\hat{x}}_{i}$, for $i=1\ldots n_{0}$, represents the normalized height of the building floors with $n_{0}$ levels.

It is important to note that the response variance used in Equation (28) is not explicitly a function of the measurements once the GPR hyperparameters are fixed. Therefore, it can be evaluated directly after the GPR model is trained. However, the training process itself (i.e., minimizing Equation (26)) depends on the sensor locations and requires measurements.

If the building response is available over the entire domain (i.e., at all floors), the optimal sensor placement and the GPR training can be solved iteratively until the optimal sensor locations $x^{*}$ coincide with the locations used for GPR training $\bar{x}$. This situation is possible when an accurate numerical model of the building is available to simulate responses at all floors, or when the building is temporarily densely instrumented and only a subset of sensors is to remain permanently. If neither of these conditions is met, the sensor locations obtained from minimizing Equation (29) represent a suboptimal solution.

### Optimization Process

This section describes the procedure for solving Equation (29), whether it is used within an iterative GPR training framework or as a single-step process.

In practical applications, some sensors are typically placed at critical locations for reasons other than minimizing response prediction uncertainty (e.g., sensors at the base of the building). Therefore, the vector $\bar{x}$ is divided into two groups: ${\bar{x}}^{f}$, representing the fixed sensor locations, and ${\bar{x}}^{r}$ representing the locations of the $n_{r}$ sensors that can vary during the optimization.

**Figure 2:**
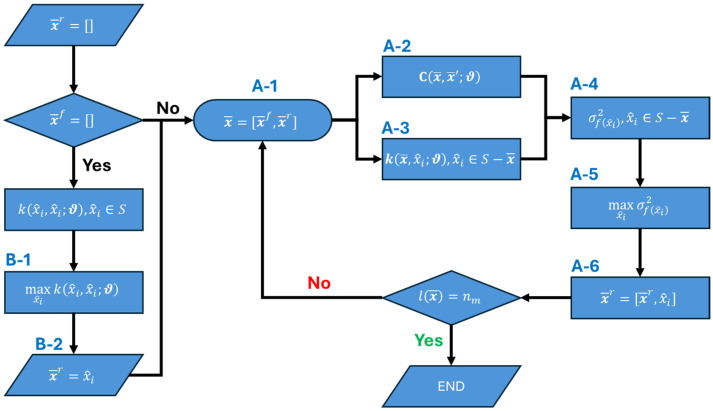
A flowchart illustrating Step 1 (greedy search) of the optimal sensor placement framework.

The optimization is carried out in two steps:

Step 1—Greedy Search: In this step, a greedy search is conducted to obtain an approximate solution for ${\bar{x}}^{r}$. Figure 2 presents a flowchart for Step 1, the steps of which are described as follows. Considering $\bar{x}={\bar{x}}^{f}$ (A-1), the covariance matrix is first calculated according to Equation (21) (A-2). Next, the kernel function is evaluated at all $n_{0}$ floors using Equation (22) (A-3). The response uncertainty at all floors, except those in $\bar{x}$, is then calculated through Equation (25) (A-4). The floor with the maximum uncertainty is selected as a candidate for instrumentation (A-5). This floor is added as the first element of ${\bar{x}}^{r}$ and removed from the list of future candidates (A-6). The process is repeated $n_{r}$ times, each time updating the covariance structure based on the previously selected locations. If no pre-selected sensor locations exist (i.e., ${\bar{x}}^{f}=\emptyset$), the location with the maximum prior variance (B-1) is selected as the first element of ${\bar{x}}^{r}$ (B-2).

Step 2—Continuous Optimization: Step 2 is a continuous optimization problem carried out in MATLAB (version 24.1.0) [35] using the derivative-free search method fminsearch [36]. To enforce bound constraints ($0\leq \bar{x}\leq 1$), a transformation is applied to convert the bounded problem into an unconstrained one [37]. The optimization starts from the approximate solution obtained in Step 1 and updates the entire vector ${\bar{x}}^{r}$ at each iteration to minimize the objective function defined in Equation (29). Although the problem is inherently discrete (floor numbers), the optimization is performed in the continuous domain, and the final solution is mapped to the nearest physical floors. During this step, no measurements are required, and $\bar{x}$ can be treated as a continuous parameter varying between 0 and 1.

Iterative GPR-OSP Procedure: As discussed earlier, the optimal sensor locations may be suboptimal if the GPR model is trained using a different set of sensor locations. If measurements are available at all floors, Steps 1 and 2 can be repeated using a GPR model retrained with data from the updated sensor locations $x^{*}$. This iterative process continues until the sensor locations used for GPR training match those obtained from the OSP procedure. In this study, the normalized Euclidean distance defined below is used as the convergence criterion:

(30) $$E=\frac{1}{n_{r}}\left\|{\left(x^{*}\right)}_{iter+1}-{\left(x^{*}\right)}_{iter}\right\|.$$

The iteration stops when this value becomes less than 0.001.

**Figure 3:**
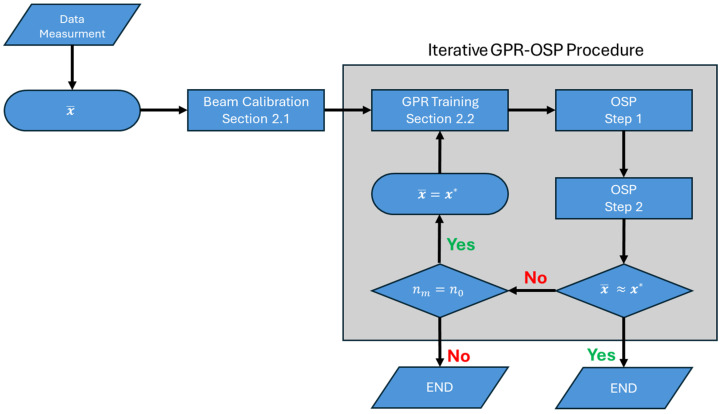
A flowchart illustrating the entire optimal sensor placement framework.

Figure 3 presents a flowchart of the entire process for the optimal sensor placement procedure, including the iterative approach whose details are provided as follows: As shown in this figure, given data from measurement or simulation, the beam calibration is carried out using an initial sensor placement ($\bar{x}$). Then, the residuals at these locations are calculated and used to train the GPR model. The trained GPR is used within Step 1 of the OSP process (Figure 2) to find the approximate optimal locations. These initial locations are used as a starting point to carry out Step 2 of the optimization. If these optimal locations ($x^{*}$) match the locations used for GPR training, the process stops. If not, and if there are measurements/simulations at all floors, these new locations are used to retrain the GPR model and the process repeats. This loop continues until the error defined in Equation (30) is less than 0.001. The iterative OSP process converges after only a few (fewer than 10) iterations and takes less than a minute on an ordinary computer.

Although the estimated response uncertainty $\sigma_{f\left(\hat{x}\right)}^{2}$ is not explicitly time-dependent, the GPR hyperparameters ϑ may vary with time if the full time history is used for training, as done in [11]. To eliminate this time dependency within the optimal sensor placement framework, the following time-independent residual is used for GPR training instead of the instantaneous residual:

(31) $$\bar{\bar{y}}={\left[\begin{array}{cccc} {\bar{\bar{y}}}_{1} & {\bar{\bar{y}}}_{2} & \cdots & {\bar{\bar{y}}}_{n_{d}} \end{array}\right]}^{T},$$

where

(32) $${\bar{\bar{y}}}_{i}=\sqrt{\frac{\sum_{n=1}^{N}{\left({\bar{y}}_{i}[n]\right)}^{2}}{N}}.$$

This formulation allows the OSP process to rely on a single set of hyperparameters representative of the entire response history.

## 4. Verification

**Figure 4:**
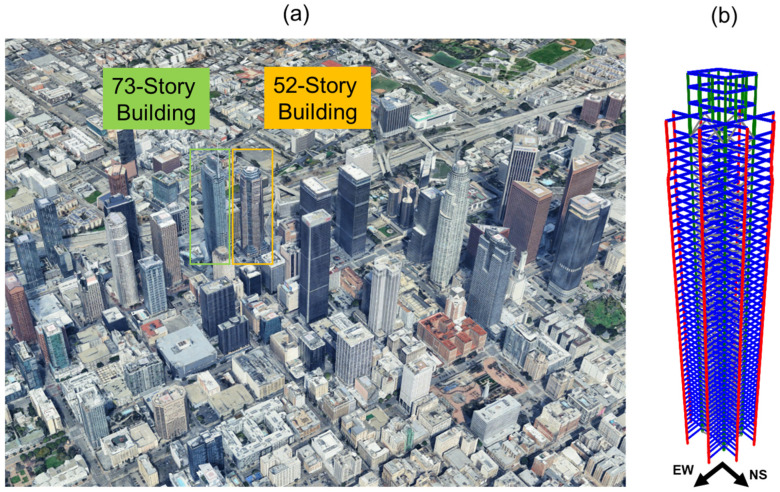
(**a**) Google Earth view of Downtown Los Angeles and (**b**) Finite Element model of the 52-story building.

To verify the proposed optimal sensor placement method, the numerical model used in [11] is employed here. This model is a Finite Element (FE) representation of a 52-story building located in downtown Los Angeles (see Figure 4). The building is instrumented by CSMIP (Station No. 24602) and has been the subject of several studies [38,39]. The lateral load-resisting system consists of a concentrically braced steel frame. The building includes multiple underground levels; however, it is assumed to be fixed at the first basement level, resulting in a total of 53 stories (54 possible instrumentation levels). The building responses in the East–West (EW) direction under the six earthquakes listed in Table 1 are used in this section. Similar to [11], the frequency of interest is limited to 10 Hz, which covers the first 10 modes of the building. For the dynamic analysis, a 2% damping ratio is assumed for all modes.

While both displacement and acceleration formulations are presented in this paper, the results are shown only for acceleration responses, which are the direct measurements obtained in practice.

The results of the beam calibration for all earthquakes are presented in Table 1. To obtain these values, acceleration responses at Levels 1 (base), 6, 7, 8, 26, 31, 41, 46, and 54 (roof) are used for calibration. The normalized heights of these levels are 0, 0.11, 0.13, 0.14, 0.47, 0.55, 0.73, 0.82, and 1, respectively. The sensor locations at Levels 6, 7, and 8 are clearly not suitable because they are too close to one another; however, they are intentionally selected to make the optimal sensor placement problem meaningful. Contrary to our previous study [11], the fundamental natural period $T_{1}$ is assumed to be known, which is a reasonable assumption because it can be readily identified from the Fourier transform of the roof response. Therefore, only α and the modal damping ratios are identified. As a result, the differences between these results and those reported in [11] arise from this modification in the calibration process.

As shown in Table 1, α is approximately 14 (indicating a balanced contribution of flexural and shear behavior) in almost all cases, except for the 2008 Chino Hills earthquake. For this event, the second mode is the dominant mode; consequently, the calibration is more influenced by this mode, resulting in a different value of α compared to the other events. The identified large first modal damping ratio further supports this observation. The identified modal damping ratios differ from the ground-truth value (2%) because the beam model is not a perfect representation of the FE model; consequently, the damping values are updated during the beam calibration to minimize the difference between the responses of the FE model and the beam model. The reader can find more details about the modeling uncertainty in beam calibration in [40].

Having calibrated beam models, the residuals (i.e., the differences between simulated and beam responses) are computed and used in the two-step optimal sensor placement procedure shown in Figure 3. Since this is a simulated example, the response at every floor is available, allowing the iterative process to be applied. In this example, it is assumed that sensors at the lowest and highest levels are fixed and cannot be moved, i.e., ${\bar{x}}^{f}={\left[0,\;1\right]}^{T}$.

For GPR training, it is common practice to normalize the data. Thus, the vector $\bar{\bar{y}}$ is normalized as $\left(\bar{\bar{y}}-\mu_{\bar{\bar{y}}}\right)/S_{\bar{\bar{y}}}$, where $\mu_{\bar{\bar{y}}}=\left(\sum_{i=1}^{n_{d}}{\bar{\bar{y}}}_{i}\right)/n_{d}$ is the mean and $S_{\bar{\bar{y}}}=\sqrt{\sum_{i=1}^{n_{d}}{\left|{\bar{\bar{y}}}_{i}-\mu_{\bar{\bar{y}}}\right|}^{2}/\left(n_{d}-1\right)}$ is the standard deviation of the data. With this data normalization, the initial value of $\sigma_{f}^{2}$ is set to 1, and the initial correlation length $\sigma_{l}$ is set to 0.5, since the spatial domain ranges from 0 to 1. Because the measurements are high-quality (simulation data), the measurement noise variance is assumed to be very small, $\sigma_{0}^{2}=1\times 10^{-5}$.

After training the initial GPR model, Step 1 of the OSP approach—the greedy search—is conducted. Step 2 then uses the results of Step 1 as initial values. The GPR training and two-step OSP procedure are iteratively repeated until there is no significant change in $\bar{x}$ used for GPR training and in the optimal locations $x^{*}$ obtained from OSP. It is noteworthy that Step 1 in the OSP always starts with ${\bar{x}}^{r}=\emptyset$ in each iteration, regardless of previous results, to ensure convergence to the global solution. An alternative approach would be to skip Step 1 and after GPR training use the same locations for the new Step 2. However, this can lead to convergence to a local solution rather than the global optimum.

Table 2 presents the final optimal locations (normalized heights) of all nine sensors obtained for all six earthquakes. For the reader’s convenience, the initial locations are also provided. As shown, although some sensors were initially poorly distributed, the final distribution is nearly uniform, consistent with the characteristics of this building model, which is uniform along its height.

This table also indicates that the final GPR model is similar across all events, with results identical except for a minor discrepancy in the fifth sensor results for the Chino Hills and M6.4 Ridgecrest earthquakes. The estimated parameters of the final GPR models, reported in Table 3, support this observation. The last column of Table 2 shows the reduction in total uncertainty scaled by the initial uncertainty, $\left[OF\left(\bar{x}\right)-OF\left(x^{*}\right)\right]/OF\left(\bar{x}\right)$. Even small improvements in the placement of a few sensors lead to a substantial reduction in the total uncertainty of the response prediction.

**Figure 5:**
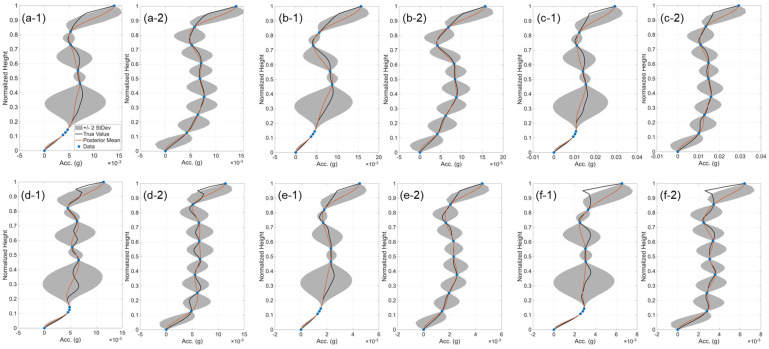
Comparison of the estimated mean residual response (Equation (32)) and its uncertainty (±2 standard deviations) with the true values for: (**1**) initial instrumentation and (**2**) optimal instrumentation, for the following earthquakes: (**a-1**,**a-2**) 1992 Landers, (**b-1**,**b-2**) 1992 Big Bears, (**c-1**,**c-2**) 1994 Northridge, (**d-1**,**d-2**) 2008 Chino Hills, (**e-1**,**e-2**) 2019 M6.4 Ridgecrest, and (**f-1**,**f-2**) 2019 M7.1 Ridgecrest.

Figure 5 shows the estimated mean (red curve) and uncertainty (gray region) of ${\bar{\bar{y}}}_{i}$ along the building height, compared with the true values (black curve) obtained from the numerical model under all six earthquake excitations. In subplots (1), the sensors (blue markers) follow the initial arrangement, resulting in regions of significant uncertainty, where the mean prediction deviates noticeably from the true values. Following the iterative OSP approach, however, the uncertainty is minimized and distributed nearly uniformly along the building height (subplots 2). This reduction in uncertainty also improves the accuracy of the predicted mean, bringing it close to the true value.

**Figure 6:**
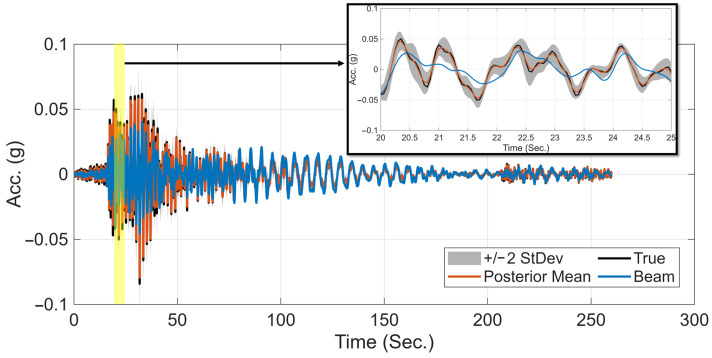
Comparison of the ground-truth acceleration response (black) at the 17th level (normalized height 0.3) with the corresponding responses from the calibrated beam model (blue) and the hybrid model (red) under the 1992 Landers earthquake. The estimation uncertainty (±2 standard deviations) is shown in gray.

Using the trained GPR model, the mean and uncertainty of the residual time history can be predicted and combined with the deterministic response from the calibrated beam model to estimate the total time-history response. Figure 6 compares the ground-truth acceleration response (black) at the 17th level (normalized height 0.3) with the response obtained from the calibrated beam (blue) and the hybrid model (red) under the 1992 Landers earthquake. The estimation uncertainty (±2 standard deviations) is shown in gray. As observed, the hybrid model prediction closely matches the ground-truth response, and the uncertainty around the prediction is very small, despite the absence of a sensor at that location.

In the example used in this section, the response of the building at every floor is available due to access to a numerical model. While it is possible to develop an accurate numerical model in real-world applications to perform optimal sensor placement, this may not always be feasible due to limited access to the information and resources required to build such models. For example, in the recent seismic instrumentation upgrade project carried out by CSMIP, neither a numerical model nor seismic data at every floor of the buildings were available. As mentioned earlier, in such scenarios, the GPR model trained using the existing data must be used for the OSP process, which may result in a suboptimal solution depending on the complexity of the structure and the number of sensors. For the example studied in this section, using the initial GPR model would lead to optimal locations (normalized heights) of 0, 0.13, 0.25, 0.38, 0.50, 0.63, 0.73, 0.86, and 1 for all events. These locations are almost identical to the optimal results shown in Table 2 (except for the sixth sensor) and achieve a similar level of reduction in total uncertainty as the optimal solution. However, this outcome is not guaranteed in every case, especially if the initial sensor layout is not adequate to properly train the GPR model.

**Figure 7:**
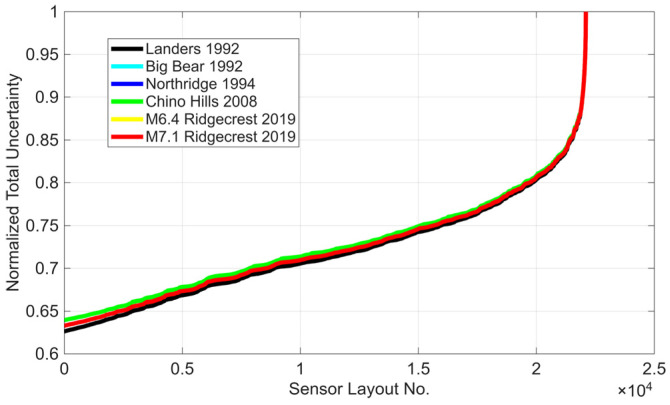
Effect of sensor distribution on the reduction of total uncertainty relative to the worst instrumentation scenario.

It is noteworthy that the entire iterative optimal sensor placement process presented above takes less than a minute on an ordinary laptop computer. In contrast, finding the optimal locations of 7 sensors out of 52 possible levels using a brute-force approach would require testing 133,784,560 distinct sensor combinations, for each of which a GPR model must be trained. While it is not feasible to perform such an exhaustive analysis to verify the optimal solution obtained in this example, it is possible to validate the results for a smaller number of sensors. Assuming that there are 5 sensors instead of 9, and that the sensors at the base and roof are fixed, there are 22,100 possible sensor combinations that can be directly analyzed (by training the GPR model and calculating the total variance). Figure 7 shows the total uncertainty for all these possible instrumentation layouts, normalized by the total uncertainty corresponding to the worst instrumentation scenario. As shown, the best instrumentation scenario (the first point in the figure) reduces the total uncertainty by approximately 37% compared to the worst instrumentation scenario (the last point in the figure) for almost all events. This optimal layout consists of sensors placed at Levels 1, 15, 28, 41, and 54 for all events, which exactly matches the results obtained using the proposed optimal sensor placement method. It is also noteworthy that, according to this brute-force analysis, the worst instrumentation scenario occurs when sensors are placed at Levels 1, 51, 52, 53, and 54.

**Figure 8:**
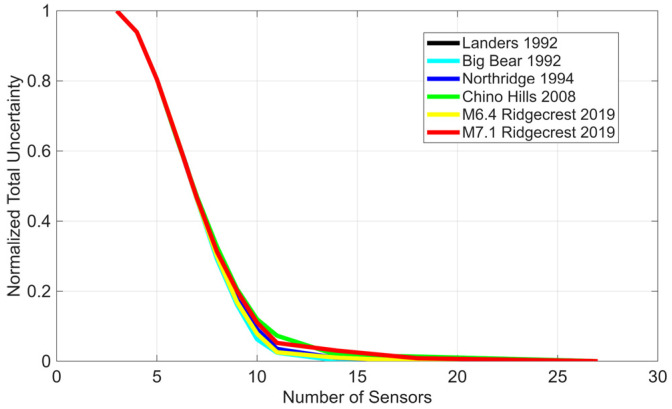
Effect of the number of sensors on the reduction of total uncertainty relative to the traditional 3-sensor instrumentation scenario.

While determining the optimal number of sensors is beyond the scope of this paper, an analysis is conducted for this numerical example to examine the reduction in total uncertainty as the number of sensors increases and they are placed at their optimal locations. Figure 8 shows the reduction in total uncertainty versus the number of sensors. The total uncertainty in this figure is normalized by the total uncertainty corresponding to the 3-sensor configuration, which is the minimum instrumentation scenario recommended in design codes (see, e.g., [41]). As shown, the uncertainty decreases approximately linearly as additional sensors are added, up to 10 sensors in this example. The behavior of the curve changes at 10 sensors because the reference data consists of 10 linearly combined modes. Theoretically, the covariance matrix would become rank-deficient, and the uncertainty would approach zero beyond this point. However, since residual data are used for training and the total uncertainty is considered, adding more sensors continues to provide additional information, although the total uncertainty beyond this point is very small compared to the 3-sensor scenario. The curve asymptotically approaches the measurement noise level.

## 5. Real-Life Applications

Validation of the proposed optimal sensor placement method would require data from a tall building with full instrumentation, which is not currently available. However, to demonstrate the application of the method and to enable assessment of the results, real earthquake data from two instrumented tall buildings are used in this section.

### 5.1. 52-Story Building

**Figure 9:**
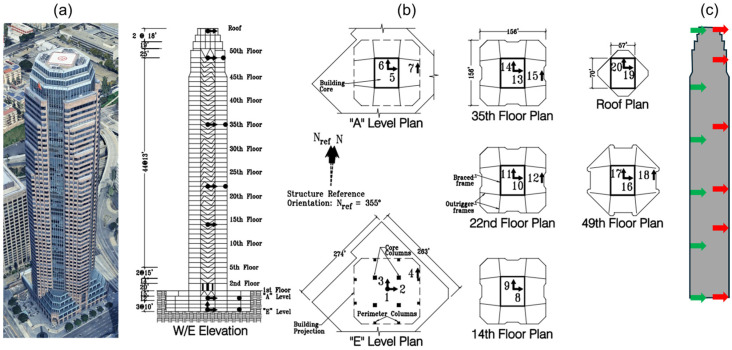
(**a**) Google Earth view, (**b**) existing instrumentation layout, and (**c**) comparison between existing (red arrows) and optimal (green arrows) sensor locations along the height of the 52-story.

The first case study considers the same 52-story building (CSMIP Station No. 24602) used in the verification section (see Figure 4a and Figure 9a). Figure 9b shows the existing instrumentation of this building. Data from sensors oriented in the East–West direction (Channels 5, 8, 10, 13, 16, and 19) are used in this study to maintain consistency with the results reported in [11]. These sensors are located on Levels 1, 15, 23, 36, 50, and 54 (counting from Level A), with normalized heights of 0, 0.27, 0.41, 0.64, 0.89, and 1, respectively.

Real earthquake data from the same events used in the verification example are also used here. The results of the beam calibration using acceleration data are reported in Table 4. Keeping the sensors at the base and roof levels fixed, the optimal sensor placement is carried out without iteration, and the results are presented in Table 5. As shown, the OSP method distributes the remaining three sensors approximately uniformly along the height, maintaining a nearly constant normalized spacing of 0.2 between adjacent sensors. This distribution is reasonable given the dynamic characteristics of this building. It is important to note that all levels are treated equally in this study, and their contributions to the total uncertainty are assumed to be the same. Because the beam model captures the primary trend of the response, the residual response is nearly uniform along the height; consequently, the uncertainty is also distributed uniformly when sensors are placed uniformly.

Similar to the verification example, nearly identical results are obtained for all earthquakes. The improvement achieved by relocating the sensors to these optimal positions is quantified as the reduction in total uncertainty relative to the existing layout. Since the sensors are already positioned close to their optimal locations, the improvement is modest, at approximately 10%. A schematic comparison between the existing and optimal sensor locations for this building is shown in Figure 9c.

### 5.2. 73-Story Building

**Figure 10:**
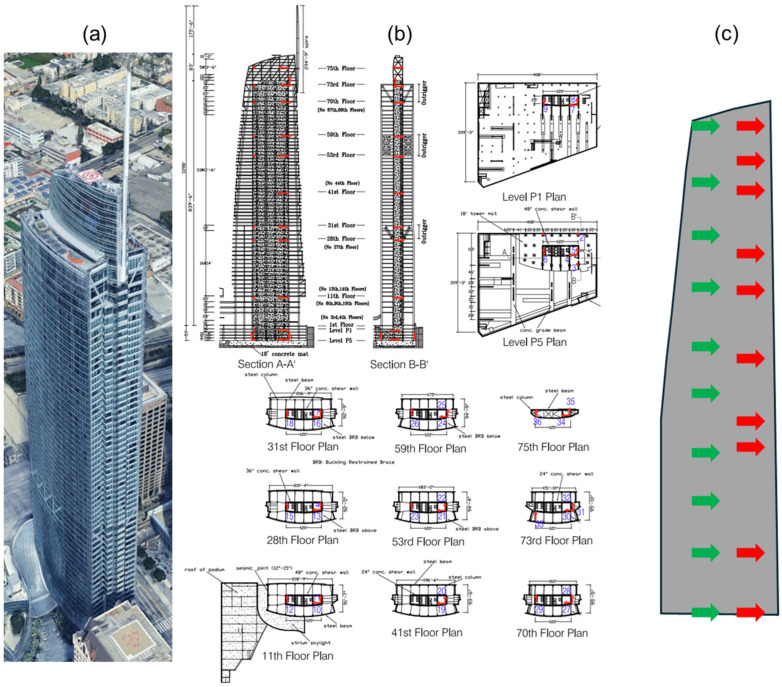
(**a**) Google Earth view, (**b**) existing instrumentation layout, and (**c**) comparison between existing (red arrows) and optimal (green arrows) sensor locations along the height of the 73-story building.

The second case study is a 73-story building (CSMIP Station No. 24660), constructed in 2017, adjacent to the 52-story building (see Figure 4a and Figure 10a). With a height of 335 m, this is presently the tallest building in California. Its lateral force–resisting system consists of core shear walls (1.2 m thick at the base, tapering to 0.6 m at the top), three outriggers with buckling-restrained braces, and two truss belts. Twenty concrete-filled steel box columns that work with the outriggers provide vertical load support. Further details can be found in [42]. The instrumentation layout of this building is shown in Figure 10b. For consistency with the results reported in [11], this study uses data from sensors oriented in the East–West direction (Channels 7, 10, 13, 16, 19, 21, 24, 27, 30, and 34). These sensors are located on Levels 1, 7, 21, 24, 34, 45, 51, 60, 63, and 68 (counting from Level P1), corresponding to normalized heights of 0, 0.13, 0.35, 0.40, 0.53, 0.67, 0.74, 0.87, 0.94, and 1.

This building has recorded four events, and data from the two 2019 Ridgecrest earthquakes are used here. For the reader’s convenience, the results of the beam calibration from [11] are reported in Table 6. The building’s lateral behavior is primarily governed by the concrete shear walls, so the calibrated beam reflects predominantly flexural behavior.

With the sensors at the base and top levels fixed, the optimal sensor placement (OSP) without iteration was performed, and the results are shown in Table 7. As observed, the OSP method distributes the remaining seven sensors almost uniformly along the height, maintaining a roughly constant normalized distance of ~0.11 between adjacent sensors. The existing instrumentation was designed to capture local behavior around the outriggers, so response estimation at non-instrumented floors is associated with large uncertainties. Redistributing the sensors uniformly results in a substantial reduction in total uncertainty—approximately 80% and 60% for the M6.4 and M7.1 Ridgecrest earthquakes of 2019, respectively. A schematic comparison of the existing and optimal sensor locations in this building is shown in Figure 10c.

## 6. Conclusions

An efficient methodology is presented in this paper to determine the optimal locations of seismic sensors along the height of buildings, aiming to minimize the uncertainty in reconstructing responses at non-instrumented floors. Installing sensors on every floor is costly and often financially unfeasible, particularly for regional-scale programs such as the California Strong Motion Instrumentation Program (CSMIP). Also, for buildings that are instrumented under seismic design codes and regulations, it is not required to instrument all floors of buildings. Building on a recently developed hybrid model–data-driven approach for estimating responses and associated uncertainties at non-instrumented floors, this study proposes a methodology to determine the optimal locations of a limited number of sensors to achieve the highest certainty in response estimation. In the two-step response reconstruction method, uncertainty arises primarily from the Gaussian Process Regression (GPR) model; therefore, the optimal sensor placement strategy identifies locations that minimize this estimation uncertainty. The methodology was thoroughly verified using a realistic finite element model of a 52-story building and subsequently applied to real data from two instrumented tall buildings, demonstrating its effectiveness in reducing uncertainty in response estimation. For the two real-life cases analyzed, the reduction in response uncertainty reached approximately 10% for the 52-story building, where existing sensors were already near-uniformly positioned. In contrast, an 80% reduction was observed for the 73-story building, as the original sensor configuration had been specifically designed to capture localized structural behavior rather than global response. This method provides a fast and efficient solution with minimum information and computational cost to plan the instrumentation layout of buildings. The method is versatile and is not limited to specific classes of buildings or vibration levels. However, it is currently designed for 2D problems where the response of the building in two directions can be analyzed separately. The extension to realistic 3D problems will be the subject of future work.

## Figures and Tables

**Table 1:** Selected earthquakes recorded by the 52-story building and the corresponding parameters of the calibrated beams.

No.	Earthquake	Year	Magnitude	Distance (km)	$T_{1}$ (s.)	α	$\xi_{1}$ (%)	$\xi_{2}$ (%)	$\xi_{3}$ (%)	$\xi_{4}$ (%)	$\xi_{5}$ (%)
1	Landers	1992	7.3 ML	169	5.45	14.11	2.01	2.68	4.61	1.81	18.57
2	BigBear	1992	6.5 ML	133	5.45	14.57	2.33	3.12	6.93	1.64	12.15
3	Northridge	1994	6.4 ML	31	5.45	14.07	2.00	2.76	6.99	1.67	13.97
4	Chino Hills	2008	5.4 Mw	47	5.45	18.41	18.97	5.00	10.65	6.66	1.95
5	Ridgecrest	2019	6.4 Mw	196	5.45	14.58	8.52	2.59	6.73	1.68	8.09
6	Ridgecrest	2019	7.1 Mw	200	5.45	14.43	2.03	2.31	6.42	1.59	12.87

**Table 2:** Optimal normalized sensor heights for the 52-story building numerical model.

Events\Normalized Heights	${\bar{x}}_{1}$	${\bar{x}}_{2}$	${\bar{x}}_{3}$	${\bar{x}}_{4}$	${\bar{x}}_{5}$	${\bar{x}}_{6}$	${\bar{x}}_{7}$	${\bar{x}}_{8}$	${\bar{x}}_{9}$	∆OF%
Initial	0	0.11	0.13	0.14	0.47	0.55	0.73	0.82	1	---
Landers, 1992	0	0.13	0.25	0.38	0.50	0.61	0.73	0.86	1	35
BigBear, 1992	0	0.13	0.25	0.38	0.50	0.61	0.73	0.86	1	32
Northridge, 1994	0	0.13	0.25	0.38	0.50	0.61	0.73	0.86	1	41
Chino Hills, 2008	0	0.13	0.25	0.38	0.48	0.61	0.73	0.86	1	51
M 6.4 Ridgecrest, 2019	0	0.13	0.25	0.38	0.50	0.61	0.73	0.86	1	39
M 7.1 Ridgecrest, 2019	0	0.13	0.25	0.38	0.48	0.61	0.73	0.86	1	38

**Table 3:** Normalized signal variance ($\sigma_{f}^{2}$) and correlation length ($\sigma_{l}$) of the trained GPR models.

GPR Parameters\Event No.	1	2	3	4	5	6
$\sigma_{f}^{2}$	0.872	0.857	0.875	0.899	0.865	0.898
$\sigma_{l}$	0.072	0.073	0.072	0.070	0.073	0.070

**Table 4:** Selected earthquakes recorded by the 52-story building and the corresponding parameters of the calibrated beam model.

No.	Earthquake	$T_{1}$ (s.)	α	$\xi_{1}$ (%)	$\xi_{2}$ (%)	$\xi_{3}$ (%)	$\xi_{4}$ (%)	$\xi_{5}$ (%)	$\xi_{6}$ (%)
1	Landers	5.89	7.62	0.99	1.45	1.76	13.16	13.53	10.44
2	BigBear	5.89	8.67	1.18	1.01	1.56	10.98	12.39	7.77
3	Northridge	5.89	11.75	3.17	1.12	5.92	2.20	19.94	11.26
4	Chino Hills	5.89	8.36	3.90	1.25	1.66	7.71	8.51	8.54
5	Ridgecrest	5.89	8.54	1.73	1.24	1.51	9.34	7.32	7.16
6	Ridgecrest	5.89	10.34	1.20	5.75	9.49	2.31	13.69	14.14

**Table 5:** Optimal normalized sensor heights for the 52-story building.

Events\Normalized Heights	${\bar{x}}_{1}$	${\bar{x}}_{2}$	${\bar{x}}_{3}$	${\bar{x}}_{4}$	${\bar{x}}_{5}$	${\bar{x}}_{6}$	∆OF%
Existing	0	0.27	0.41	0.64	0.89	1	---
Landers, 1992	0	0.20	0.39	0.59	0.78	1	11
BigBear, 1992	0	0.20	0.39	0.59	0.78	1	9
Northridge, 1994	0	0.20	0.39	0.59	0.78	1	9
Chino Hills, 2008	0	0.20	0.39	0.59	0.78	1	9
M 6.4 Ridgecrest, 2019	0	0.20	0.39	0.59	0.78	1	9
M 7.1 Ridgecrest, 2019	0	0.20	0.39	0.59	0.78	1	11

**Table 6:** Selected earthquakes recorded by the 73-story building and the corresponding parameters of the calibrated beam model.

No.	Earthquake	$T_{1}$ (s.)	α	$\xi_{1}$ (%)	$\xi_{2}$ (%)	$\xi_{3}$ (%)	$\xi_{4}$ (%)	$\xi_{5}$ (%)
1	Ridgecrest	3.57	2.58	1.27	2.20	20.15	13.30	16.64
2	Ridgecrest	3.70	3.90	1.57	5.55	2.51	2.36	1.12

**Table 7:** Optimal normalized sensor heights for the 73-story building.

Events\Normalized Heights	${\bar{x}}_{1}$	${\bar{x}}_{2}$	${\bar{x}}_{3}$	${\bar{x}}_{4}$	${\bar{x}}_{5}$	${\bar{x}}_{6}$	${\bar{x}}_{7}$	${\bar{x}}_{8}$	${\bar{x}}_{9}$	${\bar{x}}_{10}$	∆OF%
Existing	0	0.13	0.35	0.40	0.53	0.67	0.74	0.87	0.94	1	---
M 6.4 Ridgecrest, 2019	0	0.10	0.22	0.33	0.45	0.56	0.68	0.78	0.89	1	83
M 7.1 Ridgecrest, 2019	0	0.13	0.24	0.35	0.45	0.56	0.67	0.77	0.89	1	63

**Figure A1:**
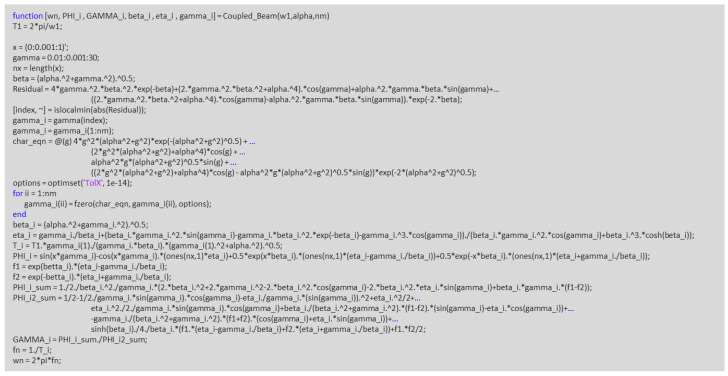
MATLAB code for calculating the modal properties of the coupled beam model. The input parameters are $\omega_{1}$, α, and $n_{m}$. The outputs are the vector of natural frequencies ($\omega_{n}$), the mode shape matrix at spatial discretization points $\phi =\left[\varphi_{1}\left(x\right)\cdots \varphi_{n_{m}}\left(x\right)\right]$, the vector of modal contribution factors ($\Gamma_{k}$), and the parameters $\beta_{k}$, $\eta_{k}$, and $\gamma_{k}$ corresponding to the $n_{m}$ modes.

**Figure A2:**
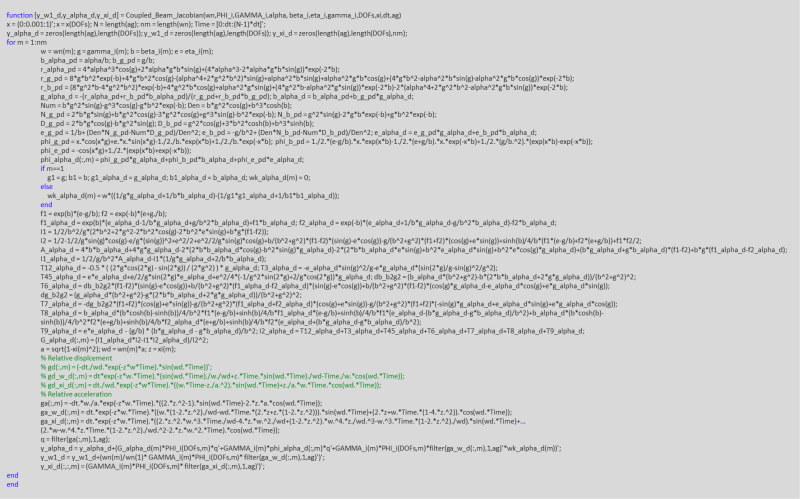
MATLAB code for calculating the elements of the Jacobian matrix. The input parameters are the vector of natural frequencies, the mode shape matrix, the vector of modal contribution factors, parameter α, and parameters $\beta_{k}$, $\eta_{k}$, and $\gamma_{k}$ corresponding to the $n_{m}$ modes, indices corresponding to the spatial locations, modal damping ratios, sampling time, and ground acceleration.

## Data Availability

These data were derived from the following resources available in the public domain: [CESMD] [https://www.strongmotioncenter.org/].

## Appendix A

The Jacobian matrix H required for the Bayesian estimation is analytically calculated as follows:

(A1) $$H=\left[\begin{array}{ccccc} \frac{\partial u_{B}\left[x_{1},1\right]}{\partial \omega_{1}} & \frac{\partial u_{B}\left[x_{1},1\right]}{\partial \alpha } & \frac{\partial u_{B}\left[x_{1},1\right]}{\partial \xi_{1}} & \cdots & \frac{\partial u_{B}\left[x_{1},1\right]}{\partial \xi_{n_{m}}}\\ \vdots & \vdots & \vdots & \ddots & \vdots \\ \frac{\partial u_{B}\left[x_{n_{d}},1\right]}{\partial \omega_{1}} & \frac{\partial u_{B}\left[x_{n_{d}},1\right]}{\partial \alpha } & \frac{\partial u_{B}\left[x_{n_{d}},1\right]}{\partial \xi_{1}} & \cdots & \frac{\partial u_{B}\left[x_{n_{d}},1\right]}{\partial \xi_{n_{m}}}\\ \frac{\partial u_{B}\left[x_{1},2\right]}{\partial \omega_{1}} & \frac{\partial u_{B}\left[x_{1},2\right]}{\partial \alpha } & \frac{\partial u_{B}\left[x_{1},2\right]}{\partial \xi_{1}} & \cdots & \frac{\partial u_{B}\left[x_{1},2\right]}{\partial \xi_{n_{m}}}\\ \vdots & \vdots & \vdots & \ddots & \vdots \\ \frac{\partial u_{B}\left[x_{n_{d}},N\right]}{\partial \omega_{1}} & \frac{\partial u_{B}\left[x_{n_{d}},N\right]}{\partial \alpha } & \frac{\partial u_{B}\left[x_{n_{d}},N\right]}{\partial \xi_{1}} & \cdots & \frac{\partial u_{B}\left[x_{n_{d}},N\right]}{\partial \xi_{n_{m}}} \end{array}\right].$$

The partial derivatives of the response with respect to the updating parameters are evaluated as follows:

(A2) $$\frac{\partial u_{B}\left[x_{i},n\right]}{\partial \omega_{1}}=\sum_{k=1}^{n_{m}}\frac{\gamma_{k}\beta_{k}}{\gamma_{1}\beta_{1}}\Gamma_{k}\phi_{k}\left(x_{i}\right)\left(\frac{\partial g_{k}^{d,a}[n]}{\partial \omega_{k}}*a_{g}[n]\right),$$

(A3) $$\frac{\partial u_{B}\left[x_{i},n\right]}{\partial \xi_{k}}=\Gamma_{k}\phi_{k}\left(x_{i}\right)\left(\frac{\partial g_{k}^{d,a}[n]}{\partial \xi_{k}}*a_{g}[n]\right),$$

(A4) $$\frac{\partial u_{B}\left[x_{i},n\right]}{\partial \alpha }=\sum_{k=1}^{n_{m}}\left\{\frac{d\Gamma_{k}}{d\alpha }\phi_{k}\left(x_{i}\right)q_{k}\left[n\right]+\Gamma_{k}\frac{d\phi_{k}\left(x_{i}\right)}{\partial \alpha }q_{k}\left[n\right]+\Gamma_{k}\phi_{k}\left(x_{i}\right)\left(\frac{\partial g_{k}^{d,a}[n]}{\partial \alpha }*a_{g}[n]\right)\right\}.$$

The partial derivatives of the IRFs (relative displacement or acceleration) with respect to modal frequencies and damping ratios are

(A5) $$\frac{\partial g_{k}^{d}[n]}{\partial \omega_{k}}=∆te^{-\xi_{k}\omega_{k}n∆t}\left\{\left(\frac{1}{\omega_{k}\omega_{d,k}}+\frac{\xi_{k}n∆t}{\omega_{d,k}}\right)\sin\left(\omega_{d,k}n∆t\right)-\frac{n∆t}{\omega_{k}}\cos\left(\omega_{d,k}n∆t\right)\right\},$$

(A6) $$\frac{\partial g_{k}^{d}[n]}{\partial \xi_{k}}=\frac{∆t}{\omega_{d,k}}e^{-\xi_{k}\omega_{k}n∆t}\left\{\left(\omega_{k}n∆t-\frac{\xi_{k}}{1-\xi_{k}^{2}}\right)\sin\left(\omega_{d,k}n∆t\right)+\frac{\omega_{k}n∆t\xi_{k}}{\sqrt{1-\xi_{k}^{2}}}\cos\left(\omega_{d,k}n∆t\right)\right\},$$

(A7) $$\frac{\partial g_{k}^{a}[n]}{\partial \omega_{k}}=∆te^{-\xi_{k}\omega_{k}n∆t}\left\{\frac{\omega_{k}\left(1-2\xi_{k}^{2}\right)}{\omega_{d,k}}-\omega_{d,k}n∆t\left[2\xi_{k}+\xi_{k}\left(1-2\xi_{k}^{2}\right)\right]\sin\left(\omega_{d,k}n∆t\right)+\left[2\xi_{k}+\omega_{k}n∆t\left(1-4\xi_{k}^{2}\right)\right]\cos\left(\omega_{d,k}n∆t\right)\right\},$$

(A8) $$\frac{\partial g_{k}^{a}[n]}{\partial \xi_{k}}=∆te^{-\xi_{k}\omega_{k}n∆t}\left\{\left(\frac{2\xi_{k}^{2}\omega_{k}^{3}n∆t-4\xi_{k}\omega_{k}^{2}}{\omega_{d,k}}+\frac{\left(1-2\xi_{k}^{2}\right)\omega_{k}^{4}\xi_{k}}{\omega_{d,k}^{3}}-\frac{\left(1-2\xi_{k}^{2}\right)\omega_{k}^{3}n∆t}{\omega_{d,k}}\right)\sin\left(\omega_{d,k}n∆t\right)+\left(2\omega_{k}-2\xi_{k}\omega_{k}^{2}n∆t-\frac{\left(1-2\xi_{k}^{2}\right)\omega_{k}^{4}\xi_{k}n∆t}{\omega_{d,k}^{2}}\right)\cos\left(\omega_{d,k}n∆t\right)\right\}.$$

The partial derivatives of the IRFs and the total derivative of mode shapes with respect to α are calculated as follows:

(A9) $$\frac{\partial g_{k}^{d,a}[n]}{\partial \alpha }=\left\{\begin{array}{c} \frac{\partial g_{k}^{d,a}[n]}{\partial \omega_{k}}\omega_{k}\left(\frac{1}{\gamma_{k}}\frac{d\gamma_{k}}{d\alpha }-\frac{1}{\gamma_{1}}\frac{d\gamma_{1}}{d\alpha }+\frac{1}{\beta_{k}}\frac{d\beta_{k}}{d\alpha }-\frac{1}{\beta_{1}}\frac{d\beta_{1}}{d\alpha }\right)\\ 0,k=1 \end{array},k\neq 1\right.,$$

(A10) $$\frac{d\phi_{k}\left(x_{i}\right)}{d\alpha }=\frac{\partial \phi_{k}\left(x_{i}\right)}{\partial \gamma_{k}}\frac{d\gamma_{k}}{d\alpha }+\frac{\partial \phi_{k}\left(x_{i}\right)}{\partial \beta_{k}}\frac{d\beta_{k}}{d\alpha }+\frac{\partial \phi_{k}\left(x_{i}\right)}{\partial \eta_{k}}\frac{d\eta_{k}}{d\alpha },$$

where

(A11) $$\frac{\partial \phi_{k}\left(x_{i}\right)}{\partial \gamma_{k}}=x_{i}\cos\left(\gamma_{k}x_{i}\right)+\eta_{k}x_{i}\sin\left(\gamma_{k}x_{i}\right)-\frac{1}{2\beta_{k}}e^{\beta_{k}x_{i}}+\frac{1}{2\beta_{k}}e^{-\beta_{k}x_{i}},$$

(A12) $$\frac{\partial \phi_{k}\left(x_{i}\right)}{\partial \beta_{k}}=\frac{1}{2}\left(\eta_{k}-\frac{\gamma_{k}}{\beta_{k}}\right)x_{i}e^{\beta_{k}x_{i}}-\frac{1}{2}\left(\eta_{k}+\frac{\gamma_{k}}{\beta_{k}}\right)x_{i}e^{-\beta_{k}x_{i}}+\frac{1}{2}\frac{\gamma_{k}}{\beta_{k}^{2}}\left(e^{\beta_{k}x_{i}}-e^{-\beta_{k}x_{i}}\right),$$

(A13) $$\frac{\partial \phi_{k}\left(x_{i}\right)}{\partial \eta_{k}}=-\cos\left(\gamma_{k}x_{i}\right)+\frac{1}{2}\left(e^{\beta_{k}x_{i}}-e^{-\beta_{k}x_{i}}\right),$$

in which

(A14) $$\frac{d\gamma_{k}}{d\alpha }=-\frac{\frac{\partial r}{\partial \alpha }+\frac{\partial r}{\partial \beta_{k}}\frac{\partial \beta_{k}}{d\alpha }}{\frac{\partial r}{\partial \gamma_{k}}+\frac{\partial r}{\partial \beta_{k}}\frac{\partial \beta_{k}}{\partial \gamma_{k}}},$$

(A15) $$\frac{d\beta_{k}}{d\alpha }=\frac{\partial \beta_{k}}{d\alpha }+\frac{\partial \beta_{k}}{\partial \gamma_{k}}\frac{d\gamma_{k}}{d\alpha },$$

(A16) $$\frac{\partial \beta_{k}}{d\alpha }=\frac{\alpha }{\beta_{k}},$$

(A17) $$\frac{\partial \beta_{k}}{\partial \gamma_{k}}=\frac{\gamma_{k}}{\beta_{k}},$$

(A18) $$\frac{\partial r}{\partial \alpha }=4\alpha^{3}\cos\gamma_{k}+2\alpha \gamma_{k}\beta_{k}\sin\gamma_{k}+\left(4\alpha^{3}-2\alpha \gamma_{k}\beta_{k}\sin\gamma_{k}\right)e^{-2\beta_{k}},$$

(A19) $$\frac{\partial r}{\partial \gamma_{k}}=8\gamma_{k}\beta_{k}^{2}e^{-\beta_{k}}+\left(4\gamma_{k}\beta_{k}^{2}+\alpha^{2}\gamma_{k}\beta_{k}\right)\cos\gamma_{k}+\left(-\alpha^{4}-2\gamma_{k}^{2}\beta_{k}^{2}+\alpha^{2}\beta_{k}\right)\sin\gamma_{k}+\left(4\gamma_{k}\beta_{k}^{2}-\alpha^{2}\beta_{k}\sin\gamma_{k}-\alpha^{2}\gamma_{k}\beta_{k}\cos\gamma_{k}\right)e^{-{2\beta }_{k}},$$

(A20) $$\frac{\partial r}{\partial \beta_{k}}=\left(8\gamma_{k}^{2}\beta_{k}-4\gamma_{k}^{2}\beta_{k}^{2}\right)e^{-\beta_{k}}+4\gamma_{k}^{2}\beta_{k}\cos\gamma_{k}+\alpha^{2}\gamma_{k}\sin\gamma_{k}+\left(4\gamma_{k}^{2}\beta_{k}-\alpha^{2}\gamma_{k}\sin\gamma_{k}-2\alpha^{4}-4\gamma_{k}^{2}\beta_{k}^{2}+2\alpha^{2}\gamma_{k}\beta_{k}\sin\gamma_{k}\right)e^{-{2\beta }_{k}},$$

and

(A21) $$\frac{d\eta_{k}}{d\alpha }=\frac{\partial \eta_{k}}{\partial \gamma_{k}}\frac{d\gamma_{k}}{d\alpha }+\frac{\partial \eta_{k}}{\partial \beta_{k}}\frac{d\beta_{k}}{d\alpha },$$

(A22) $$\frac{\partial \eta_{k}}{\partial \gamma_{k}}=\frac{1}{\beta_{k}}+\frac{\frac{\partial P_{k}}{\partial \gamma_{k}}Q_{k}-P_{k}\frac{\partial Q_{k}}{\partial \gamma_{k}}}{Q_{k}^{2}},$$

(A23) $$\frac{\partial P_{k}}{\partial \gamma_{k}}=\left(2\beta_{k}\gamma_{k}+\gamma_{k}^{3}\right)\sin\gamma_{k}+\left(\beta_{k}\gamma_{k}^{2}-3\gamma_{k}^{2}\right)\cos\gamma_{k}-\beta_{k}^{2}e^{-\beta_{k}},$$

(A24) $$\frac{\partial Q_{k}}{\partial \gamma_{k}}=2\beta_{k}\gamma_{k}\cos\gamma_{k}-\beta_{k}\gamma_{k}^{2}\sin\gamma_{k},$$

(A25) $$\frac{\partial \eta_{k}}{\partial \beta_{k}}=-\frac{\gamma_{k}}{\beta_{k}}+\frac{\frac{\partial P_{k}}{\partial \beta_{k}}Q_{k}-P_{k}\frac{\partial Q_{k}}{\partial \beta_{k}}}{Q_{k}^{2}},$$

(A26) $$\frac{\partial P_{k}}{\partial \beta_{k}}=\gamma_{k}^{2}\sin\gamma_{k}+\left(\gamma_{k}\beta_{k}^{2}-2\beta_{k}\gamma_{k}\right)e^{-\beta_{k}},$$

(A27) $$\frac{\partial Q_{k}}{\partial \beta_{k}}=\gamma_{k}^{2}\cos\gamma_{k}+3\beta_{k}^{2}\cosh\beta_{k}+\beta_{k}^{2}\sinh\beta_{k}.$$

Finally, the total derivative of the modal contribution factor with respect to α can be calculated as follows:

(A28) $$\frac{d\Gamma_{k}}{d\alpha }=\frac{I_{2}\frac{dI_{1}}{d\alpha }-I_{1}\frac{dI_{2}}{d\alpha }}{I_{2}^{1}},$$

where

(A29) $$\begin{array}{c} \frac{dI_{1}}{d\alpha }=\frac{1}{2\gamma_{k}\beta_{k}^{2}}[4\beta_{k}\frac{d\beta_{k}}{d\alpha }+4\gamma_{k}\frac{d\gamma_{k}}{d\alpha }-2\left(\beta_{k}^{2}\eta_{k}\frac{d\gamma_{k}}{d\alpha }+2\beta_{k}\frac{d\beta_{k}}{d\alpha }\right)\cos\gamma_{k}+2\left(\beta_{k}^{2}\frac{d\gamma_{k}}{d\alpha }-2\beta_{k}\eta_{k}\frac{d\beta_{k}}{d\alpha }-\beta_{k}^{2}\frac{d\eta_{k}}{d\alpha }\right)\sin\gamma_{k}+\\ \left(\beta_{k}\frac{d\gamma_{k}}{d\alpha }+\gamma_{k}\frac{d\beta_{k}}{d\alpha }\right)\left(j_{1}-j_{2}\right)+\beta_{k}\gamma_{k}\frac{d\left(j_{1}-j_{2}\right)}{d\alpha }]-I_{1}\left(\frac{1}{\gamma_{k}}\frac{d\gamma_{k}}{d\alpha }+\frac{2}{\beta_{k}}\frac{d\beta_{k}}{d\alpha }\right), \end{array}$$

(A30) $$\begin{array}{c} \frac{dI_{2}}{d\alpha }=-\frac{1}{2}\frac{2\gamma_{k}\cos{2\gamma }_{k}-\sin2\gamma_{k}}{2\gamma_{k}^{2}}\frac{d\gamma_{k}}{d\alpha }-\frac{\sin^{2}\gamma_{k}}{\gamma_{k}}\frac{d\eta_{k}}{d\alpha }-\eta_{k}\left(\frac{\sin2\gamma_{k}}{\gamma_{k}}-\frac{\sin^{2}\gamma_{k}}{\gamma_{k}^{2}}\right)\frac{d\gamma_{k}}{d\alpha }+\left(\eta_{k}+\frac{\eta_{k}}{2\gamma_{k}}\sin2\gamma_{k}\right)\frac{d\eta_{k}}{d\alpha }+\\ \frac{\eta_{k}^{2}}{4}\left(\frac{2\gamma_{k}\cos{2\gamma }_{k}-\sin2\gamma_{k}}{\gamma_{k}^{2}}\right)\frac{d\gamma_{k}}{d\alpha }+\left[\left(j_{1}-j_{2}\right)\frac{d\left[\frac{\beta_{k}}{\left(\gamma_{k}^{2}+\beta_{k}^{2}\right)}\right]}{d\alpha }+\frac{\beta_{k}}{\left(\gamma_{k}^{2}+\beta_{k}^{2}\right)}\left(\frac{dj_{1}}{d\alpha }-\frac{dj_{2}}{d\alpha }\right)\right]\left(\sin\gamma_{k}-\eta_{k}\cos\gamma_{k}\right)+\frac{\beta_{k}}{\left(\gamma_{k}^{2}+\beta_{k}^{2}\right)}(j_{1}-\\ j_{2})\left(\cos\gamma_{k}\frac{d\gamma_{k}}{d\alpha }-\cos\gamma_{k}\frac{d\eta_{k}}{d\alpha }+\eta_{k}\frac{d\gamma_{k}}{d\alpha }\sin\gamma_{k}\right)-\left[\left(j_{1}+j_{2}\right)\frac{d\left[\frac{\gamma_{k}}{\left(\gamma_{k}^{2}+\beta_{k}^{2}\right)}\right]}{d\alpha }+\frac{\gamma_{k}}{\left(\gamma_{k}^{2}+\beta_{k}^{2}\right)}\left(\frac{dj_{1}}{d\alpha }+\frac{dj_{2}}{d\alpha }\right)\right]\left(\eta_{k}\sin\gamma_{k}+\eta_{k}\cos\gamma_{k}\right)+\\ \frac{\gamma_{k}}{\left(\gamma_{k}^{2}+\beta_{k}^{2}\right)}\left(j_{1}+j_{2}\right)\left(\sin\gamma_{k}\frac{d\gamma_{k}}{d\alpha }-\sin\gamma_{k}\frac{d\eta_{k}}{d\alpha }-\eta_{k}\frac{d\gamma_{k}}{d\alpha }\cos\gamma_{k}\right)+j_{1}\left(\eta_{k}-\frac{\gamma_{k}}{\beta_{k}}\right)\frac{\beta_{k}\cosh\beta_{k}-\sinh\beta_{k}}{4\beta_{k}^{2}}\frac{d\beta_{k}}{d\alpha }+\left(\eta_{k}-\frac{\gamma_{k}}{\beta_{k}}\right)\frac{\sinh\beta_{k}}{4\beta_{k}}\frac{dj_{1}}{d\alpha }+\\ \frac{\sinh\beta_{k}}{4\beta_{k}}j_{1}\left(\frac{d\eta_{k}}{d\alpha }-\frac{\beta_{k}\frac{d\gamma_{k}}{d\alpha }-\gamma_{k}\frac{d\beta_{k}}{d\alpha }}{\beta_{k}^{2}}\right)+j_{2}\left(\eta_{k}+\frac{\gamma_{k}}{\beta_{k}}\right)\frac{\beta_{k}\cosh\beta_{k}-\sinh\beta_{k}}{4\beta_{k}^{2}}\frac{d\beta_{k}}{d\alpha }+\left(\eta_{k}+\frac{\gamma_{k}}{\beta_{k}}\right)\frac{\sinh\beta_{k}}{4\beta_{k}}\frac{dj_{2}}{d\alpha }+\frac{\sinh\beta_{k}}{4\beta_{k}}j_{2}\left(\frac{d\eta_{k}}{d\alpha }+\frac{\beta_{k}\frac{d\gamma_{k}}{d\alpha }-\gamma_{k}\frac{d\beta_{k}}{d\alpha }}{\beta_{k}^{2}}\right)+\\ \eta_{k}\frac{d\eta_{k}}{d\alpha }-\left(\frac{\gamma_{k}}{\beta_{k}}\right)\left(\frac{\beta_{k}\frac{d\gamma_{k}}{d\alpha }-\gamma_{k}\frac{d\beta_{k}}{d\alpha }}{\beta_{k}^{2}}\right), \end{array}$$

in which

(A31) $$j_{1}=\left(\eta_{k}-\frac{\gamma_{k}}{\beta_{k}}\right)e^{\beta_{k}},$$

(A32) $$j_{2}=\left(\eta_{k}+\frac{\gamma_{k}}{\beta_{k}}\right)e^{-\beta_{k}},$$

(A33) $$\frac{dj_{1}}{d\alpha }=\left(\frac{d\eta_{k}}{d\alpha }-\frac{1}{\beta_{k}}\frac{d\gamma_{k}}{d\alpha }+\frac{\gamma_{k}}{\beta_{k}^{2}}\frac{d\beta_{k}}{d\alpha }\right)e^{\beta_{k}}+j_{1}\frac{d\beta_{k}}{d\alpha },$$

(A34) $$\frac{dj_{2}}{d\alpha }=\left(\frac{d\eta_{k}}{d\alpha }+\frac{1}{\beta_{k}}\frac{d\gamma_{k}}{d\alpha }-\frac{\gamma_{k}}{\beta_{k}^{2}}\frac{d\beta_{k}}{d\alpha }\right)e^{-\beta_{k}}-j_{2}\frac{d\beta_{k}}{d\alpha },$$

(A35) $$\frac{d\left[\frac{\beta_{k}}{\left(\gamma_{k}^{2}+\beta_{k}^{2}\right)}\right]}{d\alpha }=\frac{\left(\gamma_{k}^{2}+\beta_{k}^{2}\right)\frac{d\beta_{k}}{d\alpha }-\beta_{k}\left(2\beta_{k}\frac{d\beta_{k}}{d\alpha }+2\gamma_{k}\frac{d\gamma_{k}}{d\alpha }\right)}{{\left(\gamma_{k}^{2}+\beta_{k}^{2}\right)}^{2}},$$

(A36) $$\frac{d\left[\frac{\gamma_{k}}{\left(\gamma_{k}^{2}+\beta_{k}^{2}\right)}\right]}{d\alpha }=\frac{\left(\gamma_{k}^{2}+\beta_{k}^{2}\right)\frac{d\gamma_{k}}{d\alpha }-\gamma_{k}\left(2\beta_{k}\frac{d\beta_{k}}{d\alpha }+2\gamma_{k}\frac{d\gamma_{k}}{d\alpha }\right)}{{\left(\gamma_{k}^{2}+\beta_{k}^{2}\right)}^{2}}.$$

For the reader’s convenience, two MATLAB [35] codes are provided in Figure A1 and Figure A2 for calculating, respectively, the modal properties and the derivatives of the response with respect to $\omega_{1}$, α, and the modal damping ratios.
